# Development and validation of a patient-specific model to predict postoperative SIRS in older patients: A two-center study

**DOI:** 10.3389/fpubh.2023.1145013

**Published:** 2023-04-17

**Authors:** Xiaoyue Li, Yaxin Lu, Chaojin Chen, Tongsen Luo, Jingjing Chen, Qi Zhang, Shaoli Zhou, Ziqing Hei, Zifeng Liu

**Affiliations:** ^1^Department of Anesthesiology, The Third Affiliated Hospital of Sun Yat-sen University, Guangzhou, China; ^2^Big Data and Artificial Intelligence Center, The Third Affiliated Hospital of Sun Yat-sen University, Guangzhou, China; ^3^Cell-gene Therapy Translational Medicine Research Center, The Third Affiliated Hospital of Sun Yat-sen University, Guangzhou, China; ^4^Department of Anesthesiology, Yuedong Hospital, The Third Affiliated Hospital of Sun Yat-sen University, Meizhou, China

**Keywords:** nomogram, postoperative SIRS, older patients, predicting model, perioperative management

## Abstract

**Introduction:**

Postoperative systemic inflammatory response syndrome (SIRS) is common in surgical patients especially in older patients, and the geriatric population with SIRS is more susceptible to sepsis, MODS, and even death. We aimed to develop and validate a model for predicting postoperative SIRS in older patients.

**Methods:**

Patients aged ≥65 years who underwent general anesthesia in two centers of Third Affiliated Hospital of Sun Yat-sen University from January 2015 to September 2020 were included. The cohort was divided into training and validation cohorts. A simple nomogram was developed to predict the postoperative SIRS in the training cohort using two logistic regression models and the brute force algorithm. The discriminative performance of this model was determined by area under the receiver operating characteristics curve (AUC). The external validity of the nomogram was assessed in the validation cohort.

**Results:**

A total of 5,904 patients spanning from January 2015 to December 2019 were enrolled in the training cohort and 1,105 patients from January 2020 to September 2020 comprised the temporal validation cohort, in which incidence rates of postoperative SIRS were 24.6 and 20.2%, respectively. Six feature variables were identified as valuable predictors to construct the nomogram, with high AUCs (0.800 [0.787, 0.813] and 0.822 [0.790, 0.854]) and relatively balanced sensitivity (0.718 and 0.739) as well as specificity (0.718 and 0.729) in both training and validation cohorts. An online risk calculator was established for clinical application.

**Conclusion:**

We developed a patient-specific model that may assist in predicting postoperative SIRS among the aged patients.

## Introduction

Postoperative Systemic inflammatory response syndrome (SIRS) occurs in over 80% of surgical intensive care unit (ICU) patients (1), and approximately one-third of the SIRS patients develop severe sepsis and septic shock (1), causing severe complications that include multiple organ dysfunction syndrome (MODS) and increased postoperative mortality (2, 3). Notably, it was reported that the incidence of SIRS is significantly higher in patients older than 75 years than in those younger than 40 years of age (4), and the geriatric population with SIRS is more susceptible to sepsis, MODS, and death due to various age-related organ dysfunctions, preexisting comorbidities, and limited physiologic reserve to cope with general anesthesia-related hemodynamic changes (5), which brings formidable healthcare challenges in the context of global aging (6). Early prediction of postoperative SIRS is extremely important for perioperative management to improve the older patients’ prognosis, and clinicians can intervene early to reduce the risk of serious complications in patients, as well as reducing the burden of health care system (7).

Multiple risk factors have been identified to facilitate the prediction of postoperative SIRS (8–10). Mehmet et al. reported that the preoperative platelet-to-lymphocyte ratio was an effective and inexpensive biomarker to predict postoperative SIRS (10) and Tang et al. reported that both lymphocyte to monocyte ratio and neutrophil to lymphocyte ratio were effective predictors of SIRS after percutaneous nephrolithotomy (11). Wang et al. also developed a nomogram for the prediction of SIRS after transrectal ultrasound-guided prostate biopsy (12). However, the accuracy and specificity of these risk factors and models are quite limited due to different populations, operation types, and age groups, and there is no evidence that they can be generalized to the older population. To date, an effective and practical prediction model for postoperative SIRS in older patients has not yet been available.

The goal of our study was to develop and validate an individualized predictive model for postoperative SIRS in older patients. We hope to use routinely measured preoperative and intraoperative variables to create a predictive model that could be easily implemented in clinical practice to help anesthesiologists and clinicians identify the older patients with high risk of SIRS and implement early intervention to prevent SIRS and subsequent severe septic shock and other fatal complications.

## Methods

This study was approved by the Institutional Ethics Committee and was censored on 18 May 2019 (No.[2019]02–609-01). The requirement for informed consent and clinical trial registration were waived by the committee. This manuscript adheres to the applicable TRIPOD guidelines according to Type 2b (13).

### Data extraction and study population

In this retrospective study, data of patients aged ≥65 years who underwent surgery in two centers of the Third Affiliated Hospital of Sun Yat-sen University (Guangzhou, China) from January 2015 to September 2020 were retrieved from the Electronic Health Record (EHR) systems. The exclusion criteria included: (1) patients with preoperative SIRS; (2) patients who underwent topical, local, nerve block, or combined spinal epidural anesthesia; (3) patients whose total intraoperative infusion volumes, fluid losses, or ASA classifications were not recorded. The detailed description of the exclusion criteria was shown in Supplementary Table S1. The patients spanning from January 2015 to December 2019 were enrolled in the training cohort, whereas those from January 2020 to September 2020 comprised the temporal validation cohort.

### Definition of postoperative SIRS

A case definition of postoperative SIRS was met when a patient exhibited two of the following four criteria within 7 days after surgery according to the American College of Chest Physicians in 2003 (1, 14, 15) body temperature ≥ 38°C or < 36°C, (2) heart rate ≥ 90 beats/min, (3) respiratory rate ≥ 20 breaths/min or arterial carbon dioxide tension <32 mmHg and (4) leucocyte count ≥12 × 10^9^/L or < 4 × 10^9^/L.

### Variable definition

We used descriptive statistics to characterize patients in the training and validation cohorts, both with and without SIRS. Eighteen variables that had been reported to be SIRS risk or predictive potential (16–23), or were thought to be clinically relevant with SIRS by expert anesthesiologists, were selected from the EHR in the study. These included demographic variables such as age and gender; comorbid conditions, including diabetes and hypertension; smoking history; preoperative laboratory variables; preoperative condition, including preoperative fever, and ASA classification, intraoperative events including total infusion volume, total fluid loss, blood loss, surgical duration, and postoperative ICU admission. The detailed definition of variables was shown in Supplementary Table S2.

### Variable selection

For univariate selection, we used a resampling technique with a 10-fold cross-validation with five replications. We built two logistic regression models for each variable: a null model containing only the intercept term and a model with a single predictor. The areas under receiver operating characteristic (AUC) of each model were calculated, and the AUC differences between models was compared (24). This analysis was performed for informative reasons but not used for predictor selection (25).

To determine the magnitude of the influence of each variable on the prediction results, we calculated the permutation importance based on the random forest model fit, together with the feature importance weights and cumulative weights (26, 27) (detailed in Supplementary method). To ensure the stability of the model building, we performed correlation analysis on continuous variables to reduce the effect of high covariance of variables with the criterion of correlation coefficient *r* < 0.75.

For the selection of the final model combination, we then use brute force search (detailed in Supplementary method) to traverse all possible model combinations in the training cohort. The variable combinations with the highest AUC were finally selected as the optimal model by modeling them separately with logistic regression.

### Model development, evaluation, and external validation

Based on logistic regression model, we constructed a nomogram to facilitate clinical decision-making. We evaluated the model effect on the training cohort using balanced cutoff, then apply this cutoff to the validation cohort for external validation.

### Online application

Based on the nomogram scoring system for postoperative SIRS prediction, further we developed a flexible application of the online web calculator (AID Cloud Technology Co., Ltd., China). Users can access the webpage and calculate the predicted value of the probability of postoperative SIRS to obtain the risk group immediately.

### Statistical analysis

For continuous variables, data are presented as mean (standard deviation) or median (interquartile range), whereas categorical variables are described by frequency. *T*-test was used for comparison between two groups of normal distribution measurement data, wilcoxon nonparametric test was used for comparison between non-normal distribution measurement data, and chi-square test was used for comparison between groups for qualitative data. Missing data were imputed using the mean for continuous variables and the mode for categorical variables. Before modeling, the raw data are placed on a scale of approximate symmetry of distribution using the Yeo-Johnson transformation and are data-centered and normalized. The Hosmer-Lemeshow test was used to assess the model’s goodness of fit. The nomogram was built using the lrm function of the R package rms (28). All statistical analyses were performed by using R version 3.6.2 software (Institute for Statistics and Mathematics, Vienna, Austria).^1^ All results were considered statistically significant at *p* < 0.05.

## Results

### Study cohort

**Figure 1:**
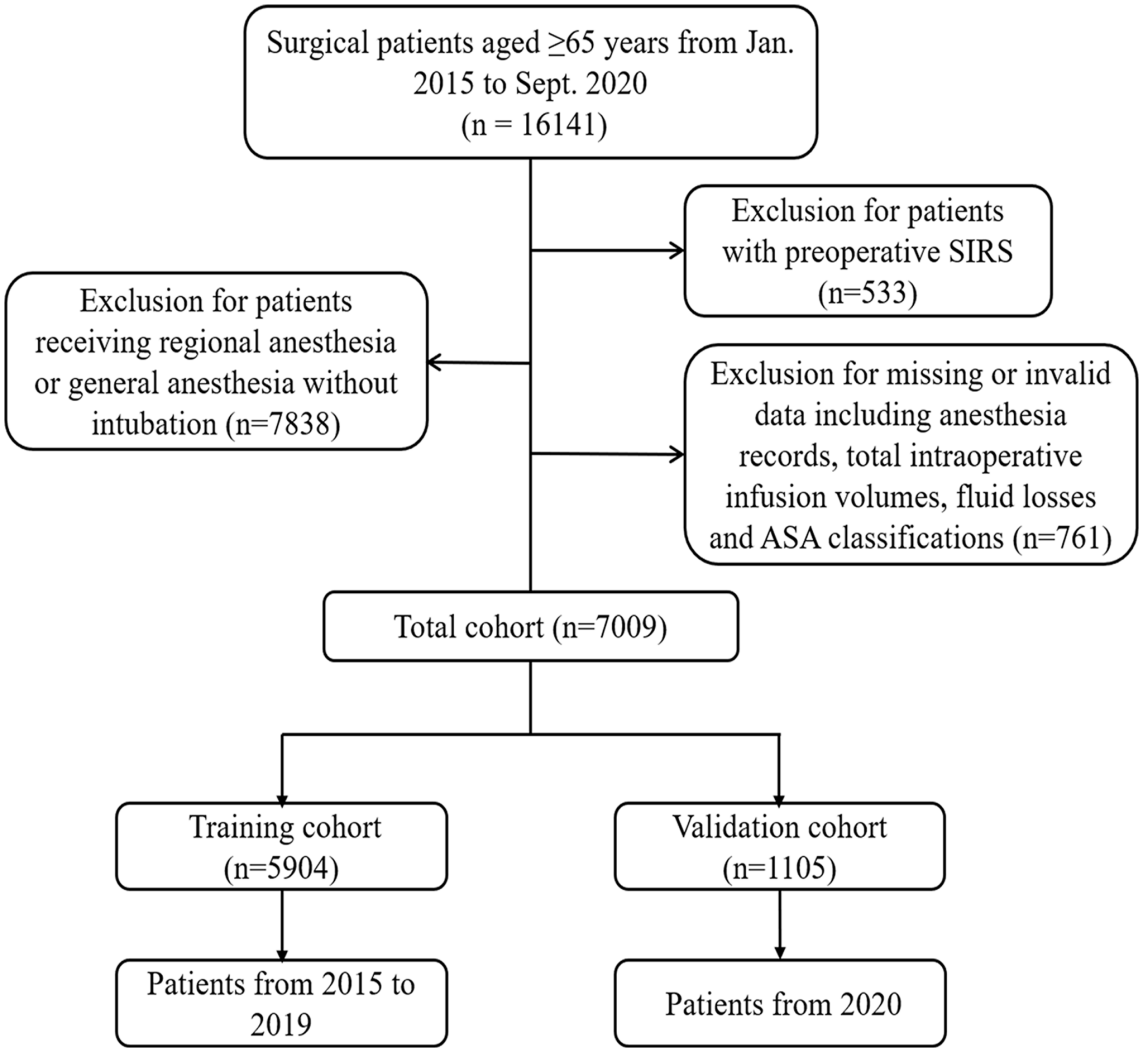
Flow chart demonstrating the patient selection process.

A total of 16,141 patients aged ≥65 years spanning the period from January 2015 to September 2020 were included. As shown in Figure 1, 533 patients with preoperative SIRS, 7838 patients receiving regional anesthesia or general anesthesia without intubation and 761 patients with missing anesthesia data or invalid data were excluded. Ultimately, 5,904 patients receiving general anesthesia with endotracheal intubation and spanning from January 2015 to December 2019 were enrolled in the training cohort, whereas 1,105 patients from January 2020 to September 2020 comprised the temporal validation cohort.

### Characteristics of training and validation cohorts

The demographic data and clinical characteristics of 7,009 cases are displayed in Supplementary Table S3. Three thousand twenty-one (43.1%) patients were women, and the average age was 70.0 (67.0–75.0) years. The numbers of SIRS patients in the training and validation cohorts were 1,451 and 882, which accounted for 24.6 and 20.2%, respectively. The prevalence rates of hypertension, diabetes mellitus, and smoking history were 58.7% (4113), 30.4% (2131), and 11.4% (802) respectively. Eight hundred eighty-nine (12.7%) patients had preoperative fever and 573 (9.74%) were transferred to the ICU. Most (65.0%) patients were categorized in ASA classification I/II.

### Differences in characteristics between non-SIRS and SIRS groups

A total of 18 features were collected from each patient in the training cohort (Table 1). After comparing the characteristics of patients with or without postoperative SIRS, we found that patients who developed postoperative SIRS were older (71.0 [67.0,76.0] vs. 70.0 [67.0,75.0], *p* < 0.001, Table 1); more likely to have been assessed at ASA III/IV/V (54.3% vs. 28.0%, *p* < 0.001); and more likely to have diabetes mellitus, a history of smoking, preoperative fever, and to have undergone preoperative intubation (all *p* < 0.001, Table 1). Grade IV surgeries were more frequent in the SIRS group (74.9% vs. 55.5%, *p* < 0.001). Meanwhile, preoperative leukocyte counts and alanine aminotransferase, hs-CRP, and creatinine levels were higher in SIRS patients than in non-SIRS patients; while levels of hemoglobin and albumin were lower in SIRS patients (all *p* < 0.001). Moreover, SIRS patients had larger intraoperative infusion, fluid loss, and blood loss volumes; and longer surgical durations than those of non-SIRS patients (all *p* < 0.001).

**Table 1:** Patient characteristics of non-SIRS and SIRS groups.

Variables	Training cohort^1^			Validation cohort^1^		
	Non-SIRS 4453 (75.4)	SIRS 1451 (24.6)	*p*-value	Non-SIRS 882 (79.8)	SIRS 223 (20.2)	*p*-value
Age^2*^, y	70.0 [67.0, 75.0]	71.0 [67.0, 76.0]	<0.001	70.0 [67.0, 74.0]	71.0 [67.0, 75.5]	0.035
Gender^1*^			<0.001			0.748
Female	2034 (45.7)	542 (37.4)		358 (40.6)	87 (39.0)	
Male	2,419 (54.3)	909 (62.6)		524 (59.4)	136 (61.0)	
Hypertension^1#^			0.403			0.764
No	1756 (39.4)	590 (40.7)		436 (49.4)	114 (51.1)	
Yes	2,697 (60.6)	861 (59.3)		446 (50.6)	109 (48.9)	
Diabetes mellitus^1*^			<0.001			0.031
No	2,997 (67.3)	860 (59.3)		823 (93.3)	198 (88.8)	
Yes	1,456 (32.7)	591 (40.7)		59 (6.69)	25 (11.2)	
History of smoking^1*^			<0.001			1.000
No	3,927 (88.2)	1,215 (83.7)		850 (96.4)	215 (96.4)	
Yes	526 (11.8)	236 (16.3)		32 (3.63)	8 (3.59)	
ASA classification^1*^			<0.001			<0.001
I/II	3,204 (72.0)	663 (45.7)		608 (68.9)	83 (37.2)	
III/IV/V	1,249 (28.0)	788 (54.3)		274 (31.1)	140 (62.8)	
Preoperative fever^1*^			<0.001			0.002
No	3,971 (89.2)	1,067 (73.5)		870 (98.6)	212 (95.1)	
Yes	482 (10.8)	384 (26.5)		12 (1.36)	11 (4.93)	
ICU admission^1*^			<0.001			<0.001
No	4,326 (97.1)	1,005 (69.3)		863 (97.8)	132 (59.2)	
Yes	127 (2.85)	446 (30.7)		19 (2.15)	91 (40.8)	
WBC^2*^, ×10^9/L	6.34 [5.17, 7.91]	7.04 [5.47, 9.32]	<0.001	6.46 [5.16, 8.08]	7.66 [5.95, 11.4]	<0.001
Total volume of infusion^2^, mL	1,500 [1,000, 2,200]	2,200 [1,500, 3,200]	<0.001	1,500 [1,000, 2,200]	2,112 [1,512, 3,050]	<0.001
ALT^2*^, U/L	17.0 [13.0, 25.0]	19.0 [13.0, 30.0]	<0.001	17.0 [13.0,25.0]	17.0 [12.8, 28.0]	0.742
hs-CRP^2*^, mg/L	6.34 [5.11, 7.97]	7.10 [5.47, 9.60]	<0.001	6.50 [5.10, 8.31]	7.80 [6.01, 11.8]	<0.001
Albumin^2*^, g/L	40.3 [37.2, 43.2]	38.2 [34.6, 41.5]	<0.001	39.9 [36.3, 42.9]	37.8 [33.0, 41.5]	<0.001
Creatinine^2*^, μmol/L	74.0 [61.0, 89.0]	78.0 [63.0, 96.0]	<0.001	71.0 [60.0, 86.0]	74.0 [56.0, 98.5]	0.294
Duration of surgery^2*^, min	125 [75.0, 200]	192 [120, 295]	<0.001	131 [77.0, 215]	193 [120, 302]	<0.001
Total volume of fluid loss^2*^, mL	350 [110, 700]	680 [350, 1,200]	<0.001	400 [100, 750]	800 [350, 1,125]	<0.001
Blood loss^2*^, mL	50.0 [10.0, 100]	100 [50.0, 200]	<0.001	40.0 [10.0, 100]	100 [50.0, 200]	<0.001
Hemoglobin^2*^, g/L	128 [116, 139]	124 [108, 137]	<0.001	126 [115, 139]	116 [100, 134]	<0.001

^1^Expressed as *n* (%). ^2^Expressed as median [Q1, Q3]. ^*^Non-SIRS group vs. SIRS group in the training cohort, *p* < 0.001. ^#^Non-SIRS group vs. SIRS group in both training cohort and validation cohort, *p* > 0.001. WBC count, white blood cell count; ALT, Alanine aminotransferase; hs-CRP, high-sensitivity c-reaction protien; SIRS, systemic inflammatory response syndrome.

### Prognosis of non-SIRS and SIRS groups

As shown in Table 2, compared with the non-SIRS group, patients in the SIRS group were significantly more likely to experience postoperative complications that included agitation and delirium, hemorrhage, pneumonia, acute kidney injury, hypotension, coma, and cardiac arrest. Patients with SIRS had a significantly worse in-hospital survival rate than patients without SIRS (all *p* < 0.001, Table 2). SIRS patients also had higher hospitalization and surgical costs, and longer postoperative and total hospital stays than those without SIRS (all *p* < 0.001, Table 2).

**Table 2:** Patients’ postoperative prognosis of non-SIRS and SIRS groups.

Variables	Total cohort (*N* = 7,009)	Non-SIRS (*N* = 5,335)	SIRS (*N* = 1,674)	*p*-value
Hemorrhage^a^	2,140 (30.50)	1,394 (26.10)	746 (44.60)	<0.001
ARDS^a^	26 (0.37)	9 (0.17)	17 (1.02)	<0.001
Pneumonia^a^	550 (7.85)	169 (3.17)	381 (22.80)	<0.001
Acute pulmonary embolism^a^	15 (0.21)	5 (0.09)	10 (0.60)	0.001
Cardiac arrest^a^	91 (1.30)	19 (0.36)	72 (4.30)	<0.001
Hypotension^a^	187 (2.67)	73 (1.37)	114 (6.81)	<0.001
Agitation and delirium^a^	187 (2.67)	51 (0.96)	136 (8.13)	<0.001
Coma^a^	188 (2.68)	18 (0.34)	170 (10.20)	<0.001
Mortality during hospitalization^a^	70 (1.00)	16 (0.30)	54 (3.23)	<0.001
ICU admission^a^	683 (9.74)	146 (2.74)	537 (32.10)	<0.001
Acute kidney injury^a^	223 (3.18)	93 (1.74)	130 (7.77)	<0.001
Postoperative hospital stay^b^	8.00 [5.00, 11.00]	7.00 [4.00, 10.0]	11.0 [8.00, 19.0]	<0.001
Total hospital stay^b^	15.0 [10.0, 22.0]	13.0 [9.00, 19.0]	21.0 [14.0, 31.0]	<0.001
Total cost^b^	55,499 [29,795, 83,605]	47,077 [25,392, 71,091]	89,500 [59,959, 136,333]	<0.001
Costs of surgery^b^	5,780 [3,679, 8,389]	5,300 [3,340, 7,902]	7,230 [4,877, 9,816]	<0.001

^a^Expressed as No. (%). ^b^Expressed as median [Q1, Q3].

### Variable selection

The results of the univariate analysis showed that all 18 candidate variables were statistically different (all *p* < 0.05) (Supplementary Table S4). We then constructed a random forest-based permutation to calculate the percentage importance weight of variables for each indicator, and calculated the cumulative weights after sorting from largest to smallest, and identified the top 8 variables with the highest weights as candidates (Supplementary Table S5). We believe that the cumulative importance weights of these 8 variables are high enough to explain nearly 90% of the outcomes (Supplementary Figure S1A).

To ensure the stability of the model construction, we conducted correlation analysis on the continuous variables and excluded the “Total volume of fluid loss” with high covariance (*r* > 0.75, Supplementary Figure S2). After that, we searched for the optimal combination of the remaining seven candidate variables by the brute force search algorithm, and the results showed that the six-variables combination had the highest AUC (Supplementary Figure S1B).

Our six-variables combination model included preoperative fever, preoperative albumin level, ASA classification, total intraoperative infusion volume, surgical duration, and postoperative ICU admission. These variables were used in subsequent studies to construct logistic regression models and in the development of nomograms and online risk calculators.

### Model construction and external validation

The predicted risk probability of postoperative SIRS can be calculated by the following model: Predicted Probability = 1/ (1 + e ^ Linear Predictor). In which, Linear Predictor = (−1.891) + (−0.177)*Albumin *+*0.178*Duration of surgery *+*0.517*ASA classification *+*0.867*Fever before surgery +2.251*ICU admission +0.534*Total volume of infusion. The cut-off value for the high-risk and low-risk groups is 0.216 based on a balance of sensitivity and specificity, which were shown in Supplementary Table S6.

**Figure 2:**
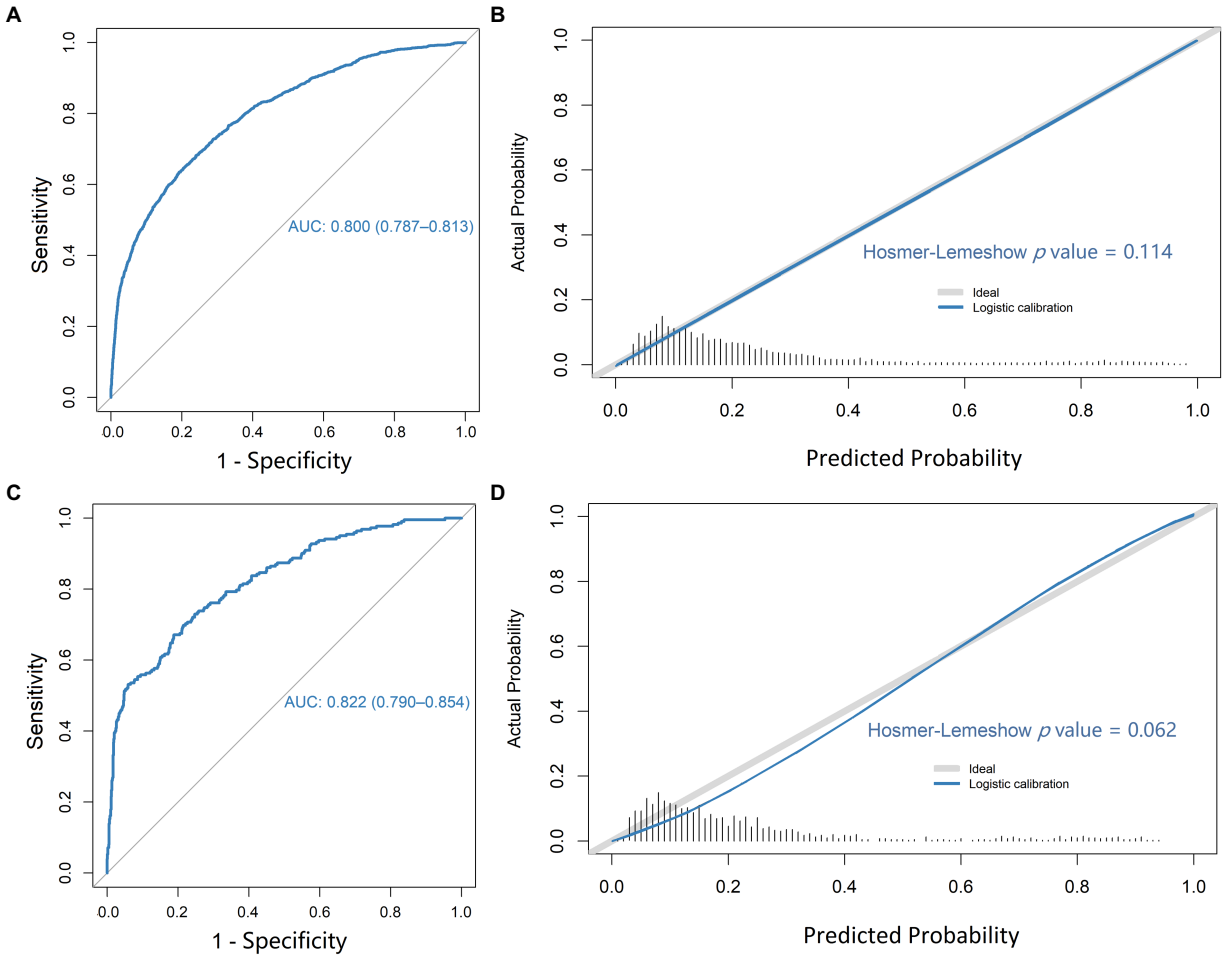
ROC plot and calibration of the logistic regression model. ROC plot **(A)** and calibration curve **(B)** for predicting patient SIRS in the training cohort; ROC plot **(C)** and calibration curve **(D)** for predicting patient SIRS in the validation cohort.

In the training cohort, the logistic regression model established by using the 6 selected predictors had a high AUC (0.800 [95% CI, 0.787–0.813]) to discriminate individuals with SIRS from those with non-SIRS, with a sensitivity of 71.8% and specificity of 71.8% (Figures 2A,B and Supplementary Table S6). The result of external validation showed that the AUC of the model was 0.822 (95% CI, 0.790, 0.854), with a sensitivity of 73.9% and specificity of 72.9% (Figures 2C,D and Supplementary Table S6). In addition, the Hosmer-Lemeshow test *p*-values of the training set and the validation set were 0.114 and 0.062 respectively, and both greater than 0.05, indicating the good quality of the fits (Figures 2B,D). The decision curve analysis (DCA) of the training cohort also showed that our model has good clinical utility (Supplementary Figure S3).

In addition, we also used the six-variable model to analyze the validation cohort hierarchically based on the potential confounding factors of SIRS. We found no significant differences between subgroups of age, gender, diabetes mellitus, hypertension, blood loss and type of surgery (Supplementary Table S7), indicating that the predictive performance of the developed model in each subgroup was relatively stable, and illustrating its high-accuracy and generalizability in each subgroup.

### Predictive nomogram and online risk calculator

**Figure 3:**
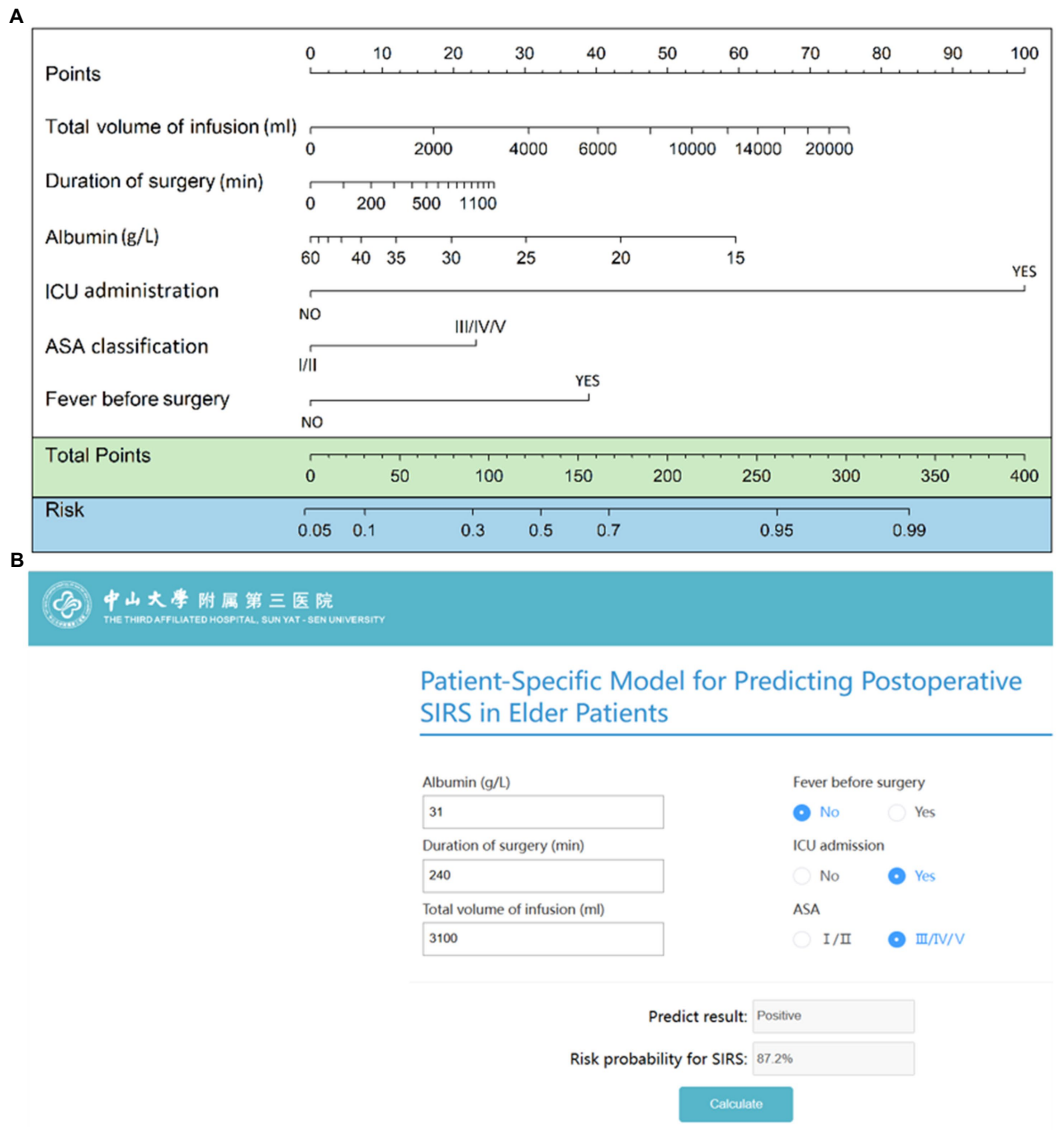
Nomogram and browse-based calculator to predict postoperative SIRS in older patients. **(A)** The nomogram to predict postoperative SIRS was created based on 6 independent feature variables, including preoperative fever, preoperative serum albumin level, ASA classification, total intraoperative infusion volume, surgical duration, and postoperative intensive care unit admission. **(B)** Browse-based calculator that can be used to help make clinical decisions regarding the potential risk of postoperative SIRS in older patients. The browse-based tool can be visited at http://wb.aidcloud.cn/zssy/SIRS.html.

Based on the final regression analysis, a nomogram was constructed that incorporated the 6 significant risk factors for predicting postoperative SIRS (Figure 3A). As reported previously (29), each variable corresponding to the nomogram was scored on a point scale axis based on its contribution to our logistic regression model. The total points that corresponded to the risk of postoperative SIRS could be calculated easily that corresponded to the risk of postoperative SIRS could be calculated easily by adding each single score. An online risk calculator (Figure 3B) to further facilitate external validation can be accessed at http://wb.aidcloud.cn/zssy/SIRS.html.

## Discussion

Because of the continuum between different stages of the inflammatory response from SIRS to sepsis and septic shock (30) and the consensus definition of severe sepsis requires signs that meet criteria for SIRS (31), early diagnosis of postoperative SIRS is critical to initiate timely interventions to prevent septic shock and improve clinical prognosis in older patients. In this study, we developed and validated the model according to type2b in TRIPOD and identified 6 feature variables that have strong independent discriminatory power for SIRS with maximal AUC values (0.800 and 0.822) and relatively balanced sensitivity (0.718 and 0.739) as well as specificity (0.718 and 0.729) in both training and validation cohorts. We also constructed a nomogram and a browse-based risk calculator based on the identified variables to distinguish older patients at high risk for postoperative SIRS and alert clinicians to provide early interventions.

Several risk factors have been associated with postoperative SIRS including mannose-binding lectin deficiency (32), high levels of circulating GM-CSF + CD4+ T cells (33), bacteriuria and renal stone size (34), diabetes mellitus, and the intraoperative use of an intra-aortic balloon pump (35). However, their predictive values are limited because measurements of these parameters are generally not available or not easily obtainable in routine testing, or only pertain to particular surgical operations. These limitations preclude their general application to the geriatric population. In our predictive model, preoperative indicators included the preoperative fever, preoperative albumin level and ASA classification, can be measured routinely and accurately for both the elective patients and emergency patients admitted to the hospital in China. Intraoperative indicators included total infusion volume, surgical duration, and postoperative ICU admission are also routinely recorded for every patient. Notably, postoperative ICU admission ranks first in feature importance weight in our model, which is in line with our clinical experience that ICU patients have higher incidence rate of SIRS or sepsis, adding clinical credibility to our model. Moreover, our results can also be interpreted as risk stratification of SIRS for postoperative ICU older patients, and in this population the other five feature variables in our model should be given higher priority.

Currently, most studies mainly predict the mortality and other adverse prognosis of sepsis using the SOFA or other criteria. However, as professor Simpson SQ (36) pointed out, the clear purpose of diagnostic criteria is to prompt physicians to intervene timely and our emphasis should also be placed on early diagnosis rather than on mortality prediction. The most important role of prediction model should also be the same, so we identified the SIRS criteria as the primary outcome due to its high sensitivity to sepsis (37). Although there is a tendency to apply criteria including SOFA score or quick SOFA score to identify the possibility of sepsis, SIRS criteria has demonstrated higher sensitivity compared to qSOFA score, and it has served as both useful inclusion criteria and therapeutic target of trails aiming to treat sepsis (38). Clinically, SIRS has been an acknowledged criterion that is easily to identify which prompt the physicians to notice the possibility of sepsis and prescribe tests to examine whether infection truly exists.

In addition, our model has important implications for public health policy, clinical practice, and the informed consent process. Firstly, the model is able to identify the risk of postoperative SIRS in older patients once after the surgery, thus providing them better intraoperative and postoperative management measures and medical resource allocation, and ultimately improving the prognosis of high-risk patients, especially those in surgical ICUs (39, 40). Secondly, all of the variables integrated in the predictive model are measured routinely during the perioperative management, this enhances the usability and generalizability of the model, making it easy for different regions and types of hospitals to use the model to assess patients. To further facilitate its external validation and application, we have established an online risk calculator^2^ (41), it has been accessible for all the peers in daily clinical practice.

To our knowledge, this was the first study to develop a nomogram and enables individualized prediction of the risk of postoperative SIRS in older patients. The nomogram supports real-time prediction embedded in EMR systems thus having straightforward applicability, and enabling the integration of a risk prediction tool as a clinical decision support aid in perioperative older patient care (42). Additionally, the nomogram provides an objective, data-based estimate of risk probabilities that can help patients and their families realize their disease prognosis and thus make informed consent decisions that are best for their health.

Several limitations in this study should be addressed. Firstly, the two-center retrospective study design may be prone to collection and entry bias, as well as residual confounding, although we have used a temporal external validation approach, the predictive potential of the model still need to be confirmed by prospective study and external validation in the future. Secondly, as the older patients receiving regional anesthesia in our hospital are generally in relatively good conditions and often require a short and minor operation that might have lower risk of postoperative SIRS, we only enrolled the patients with general anesthesia and endotracheal intubation in the study. Future prospective studies are needed to collect more clinical and genomic information to predict an individual patient’s predisposition to SIRS more precisely. Thirdly, the webtool does not include any of the data bounds that the nomogram was built on and the tool has not been tested outside of the original data bounds, but the data bounds of the continuous variables included in the nomogram are very large, including albumin (15–60 g/L), duration of surgery (0–1,400 min), total volume of infusion (0–22,000 mL), which were thought to have included the vast majority of clinical cases.

## Conclusion

We have developed and validated an effective model for predicting the risk of postoperative SIRS in older patients. Based on the model, we constructed a practical nomogram that exhibits excellent calibration. This nomogram could enable anesthesiologists and clinicians to make individualized predictions of each patient’s probability of postoperative SIRS and to improve treatment recommendations for older patients.

## Data availability statement

The original contributions presented in the study are included in the article/Supplementary material, further inquiries can be directed to the corresponding authors.

## Ethics statement

This study was approved by the Institutional Ethics Committee from the two hospitals and was censored on 18 May 2019 (No. [2019]02–609-01). Written informed consent for participation was not required for this study in accordance with the national legislation and the institutional requirements.

## Author contributions

CC and TL collected the data and verified the underlying data. CC and XL drafted the article. SZ and CC conceived and designed the study. QZ, ZL, and CC revised the article. YL, JC, and ZL did the statistical analysis. ZH and CC obtained the funding for the study. ZH and SZ supervised the study. All authors read and approved the manuscript, had full access to all the data used in the study, and had final responsibility for the decision to submit for publication.

## Funding

This study was supported partly by the National Natural Science Foundation of China (Grant No. 81974296 and 82102297), Natural Science Foundation of Guangdong Province (Grant No. 2018A0303130224 and 2022A1515012603), Science and Technology Program of Guangzhou, China (Grant No. 202002020047), the Fundamental Research Funds for the Central Universities of China (Grant No. 22qntd3401), Young Talent Support Project of Guangzhou Association for Science and Technology (Grant No. QT20220101257), and Science and Technology Planning Project of Guangzhou City (Grant No. 202206080003).

## Conflict of interest

The authors declare that the research was conducted in the absence of any commercial or financial relationships that could be construed as a potential conflict of interest.

## Publisher’s note

All claims expressed in this article are solely those of the authors and do not necessarily represent those of their affiliated organizations, or those of the publisher, the editors and the reviewers. Any product that may be evaluated in this article, or claim that may be made by its manufacturer, is not guaranteed or endorsed by the publisher.

## Footnotes

^1^ http://www.r-project.org

^2^ http://wb.aidcloud.cn/zssy/SIRS.html

## Abbreviations

SIRS: systemic inflammatory response syndrome
MODS: multiple organ dysfunction syndrome
EHR: Electronic Health Record
AUCs: areas under receiver operating characteristic
WBC count: white blood cell count
ALT: alanine aminotransferase
hs-CRP: high-sensitivity c-reaction protein
