# Supplementary material for: Development and validation of a patient-specific model to predict postoperative SIRS in older patients: A two-center study
Source: Front Public Health. 2023 Apr 17;11:1145013. doi: 10.3389/fpubh.2023.1145013 (PMC10150121; doi:10.3389/fpubh.2023.1145013)
Supplement: Supplementary file 1 [file Data_Sheet_1.docx]

**Development and validation of a patient-specific model** **to predict** **postoperative SIRS in elderly patients: A two-center study**

Xiaoyue Li^1#^; Yaxin Lu^2#^; Chaojin Chen^1,3#^; Tongsen Luo^1^; Jingjing Chen^2^; Qi Zhang^3^; Shaoli Zhou^1*^; Ziqing Hei^1,4*^; Zifeng Liu^2*^

^1^Department of Anesthesiology, The Third Affiliated Hospital of Sun Yat-sen University, Guangzhou, People’s Republic of China.

^2^ Big Data and Artificial Intelligence Center, The Third Affiliated Hospital of Sun Yat-sen University, Guangzhou, People’s Republic of China.

^3^ Cell-gene Therapy Translational Medicine Research Center, The Third Affiliated Hospital of Sun Yat-sen University; Guangzhou, People’s Republic of China.

^4^Department of Anesthesiology, Yuedong Hospital, The Third Affiliated Hospital of Sun Yat-sen University, Meizhou, People’s Republic of China.

^#^These authors contributed equally to the work.

^*^Correspondence to:

Shaoli Zhou, M.D. & Ziqing Hei, M.D., Ph.D.

Department of Anesthesiology, The Third Affiliated Hospital of Sun Yat-sen University

No. 600 Tianhe Road, Guangzhou, Guangdong Province, 510630, China.

Email: [13610272308@139.com;](mailto:13610272308@139.com;) & [heiziqing@sina.com](mailto:heiziqing@sina.com)

&

Zifeng Liu, M.D.

Clinical Data Center, The Third Affiliated Hospital of Sun Yat-sen University, Guangzhou, People’s Republic of China.

Email: [liuzf@mail.sysu.edu.cn](mailto:liuzf@mail.sysu.edu.cn)

**Supplementary material**

**Supplementary method:**

1 Brute Force search:

Brute force search is a very general and straightforward problem solving technique in computer science that, unlike other techniques, relies on pure computational power and traverses every possible solution in a finite space, thus allowing for a globally optimal solution.

In this study, we use brute force search combined with 10-fold cross-validation to iterate through all combinations of variables available in the training cohort for 18 variables and model them separately using logistic regression to select the optimal combination using the mean value of AUC as an evaluation metric.

2 Permutation:

The basic principle of permutation importance is that the importance of a feature is measured by calculating the increase in the prediction error of the model after feature permutation.

In this way, a trained model is first obtained, and predictions are made by disrupting a certain column of values. The predicted and true target values are used to calculate the extent to which the loss function is elevated due to random ordering. If disordering that column of values reduces the discriminative ability of the model, the feature is considered important and the amount of decay in model performance represents the importance of the disordered column. Then the disordered column is recovered and the above operation is repeated on the next column of data until the importance of each column is calculated. Since there is randomness in extracting significance for a disordered feature, we repeat the process by disordering it several times.

Table S1. **The detailed description of the exclusion criteria**.

| **Exclusion criteria** | **Description** |
| --- | --- |
| Patients with preoperative SIRS. | The main outcome of this study is the occurrence of postoperative SIRS, and the existence of preoperative SIRS should be excluded. |
| Patients who underwent topical, local, nerve block, or combined spinal epidural anesthesia. | As the elderly patients receiving regional anesthesia in our hospital are generally in relatively good conditions and often require a short and minor operation that might have lower risk of postoperative SIRS, we only enrolled the patients with general anesthesia and endotracheal intubation in the study. |
| Patients whose total intraoperative infusion volumes, fluid losses, or ASA classifications were not recorded. | Missing data or incomplete information. |

Table S2. The detailed definition of variables.

| **Variables** | **Definition** |
| --- | --- |
| preoperative fever | The body temperature ≥38 °C three days before operation. |
| intubation before surgery | Enter the operating room with the intubation existed. |
| total infusion volume | The total amount of fluid input during the operation, including crystal solution, colloid solution, blood products, etc. |
| total fluid loss | The total amount of fluid lost during operation includes blood loss, urine volume, etc. |
| blood loss | Amount of blood lost during surgery. |
| surgical duration | The duration from the beginning of the operation to the end of the operation. |
| postoperative ICU admission | Be admitted to ICU at the end of the operation. |

Supplementary results:

Table S3. Patient characteristics in Training and Validation cohorts.

| **Variables** | **Total**  **(N=7009)** | **Training Cohort**  **(N=5904)** | **Validation Cohort**  **(N=1105)** | ***p-*value** |
| --- | --- | --- | --- | --- |
| Age ^b^ | 70.0 [67.0,75.0] | 70.0 [67.0,75.0] | 70.0 [67.0,74.0] | 0.411 |
| Gender ^a^ |  |  |  | 0.049 |
| Female | 3021 (43.1) | 2576 (43.6) | 445 (40.3) |  |
| Male | 3988 (56.9) | 3328 (56.4) | 660 (59.7) |  |
| Hypertension ^a^ |  |  |  | <0.001 |
| No | 2896 (41.3) | 2346 (39.7) | 550 (49.8) |  |
| Yes | 4113 (58.7) | 3558 (60.3) | 555 (50.2) |  |
| Diabetes mellitus ^a^ |  |  |  | <0.001 |
| No | 4878 (69.6) | 3857 (65.3) | 1021 (92.4) |  |
| Yes | 2131 (30.4) | 2047 (34.7) | 84 (7.60) |  |
| History of smoking ^a^ |  |  |  | <0.001 |
| No | 6207 (88.6) | 5142 (87.1) | 1065 (96.4) |  |
| Yes | 802 (11.4) | 762 (12.9) | 40 (3.62) |  |
| ASA classification ^a^ |  |  |  | 0.073 |
| I/II | 4558 (65.0) | 3867 (65.5) | 691 (62.5) |  |
| III/IV/V | 2451 (35.0) | 2037 (34.5) | 414 (37.5) |  |
| Fever before surgery ^a^ |  |  |  | <0.001 |
| No | 6120 (87.3) | 5038 (85.3) | 1082 (97.9) |  |
| Yes | 889 (12.7) | 866 (14.7) | 23 (2.08) |  |
| postoperative ICU admission ^a^ |  |  |  | 0.840 |
| No | 6326 (90.3) | 5331 (90.3) | 995 (90.0) |  |
| Yes | 683 (9.74) | 573 (9.71) | 110 (9.95) |  |
| WBC count ^b^, x10^9/L | 6.50 [5.23,8.25] | 6.47 [5.23,8.20] | 6.63 [5.26,8.54] | 0.019 |
| Total volume of infusion ^b^, mL | 1600 [1100,2300] | 1600 [1100,2300] | 1650 [1100,2300] | 0.669 |
| ALT ^b^, U/L | 17.0 [13.0,26.0] | 17.0 [13.0,26.0] | 17.0 [13.0,26.0] | 0.685 |
| hs-CRP ^b^, mg/L | 6.52 [5.19,8.39] | 6.49 [5.19,8.31] | 6.69 [5.22,8.85] | 0.006 |
| Albumin ^b^, g/L | 39.8 [36.5,42.8] | 39.9 [36.6,42.9] | 39.5 [35.9,42.7] | 0.011 |
| Creatinine ^b^, µmol/L | 74.0 [61.0,90.0] | 75.0 [61.0,91.0] | 71.0 [59.0,87.0] | <0.001 |
| Duration of surgery ^b^, min | 140 [83.0,226] | 140 [82.0,225] | 145 [85.0,234] | 0.256 |
| Total volume of fluid loss^b^, mL | 420 [170,830] | 412 [170,820] | 450 [150,850] | 0.984 |
| Blood loss ^b^, mL | 50 [20,100] | 50 [20,100] | 50 [10,100] | 0.007 |
| Hemoglobin ^b^, g/L | 127 [114,138] | 127 [114,139] | 125 [111,137] | 0.010 |

^a^ expressed as No. (%); ^b^ expressed as median [Q1, Q3]; WBC count, White blood cell count; ALT, Alanine aminotransferase; hs-CRP, high-sensitivity c-reaction protien; SIRS, systemic inflammatory response syndrome;

Table S4. Univariate feature screening of systemic inflammatory response syndrome using resampling methods.

| **Variables** | **Improvement** | ***p*-value** | **AUC** | **In final model** |
| --- | --- | --- | --- | --- |
| Total volume of infusion | 0.201 | <0.001 | 0.701 | YES |
| Total volume of fluid loss | 0.183 | <0.001 | 0.683 | - |
| Blood loss | 0.169 | <0.001 | 0.669 | - |
| Duration of surgery | 0.160 | <0.001 | 0.660 | YES |
| postoperative ICU admission | 0.139 | <0.001 | 0.639 | YES |
| ASA classification | 0.131 | <0.001 | 0.631 | YES |
| Albumin | 0.114 | <0.001 | 0.614 | YES |
| hs-CRP | 0.084 | <0.001 | 0.584 | - |
| WBC count | 0.081 | <0.001 | 0.581 | - |
| Fever before surgery | 0.078 | <0.001 | 0.578 | YES |
| Hemoglobin | 0.065 | <0.001 | 0.565 | - |
| Creatinine | 0.048 | <0.001 | 0.548 | - |
| Alanine aminotransferase | 0.042 | <0.001 | 0.542 | - |
| Gender | 0.042 | <0.001 | 0.542 | - |
| Diabetes mellitus | 0.040 | <0.001 | 0.540 | - |
| Age | 0.040 | <0.001 | 0.540 | - |
| History of smoking | 0.022 | <0.001 | 0.522 | - |
| Hypertension | 0.006 | 0.025 | 0.506 | - |

Abbreviations**:** WBC count, White blood cell count; hs-CRP, high-sensitivity c-reaction protein;

Table S5. The cumulative importance weights of the 18 variables.

| **Characteristics** | **Importance** | **Feature Importance Weight** | **Cumulative Feature Importance Weight** |
| --- | --- | --- | --- |
| Postoperative ICU admission | 0.071 ± 0.002 | 0.413 | 0.413 |
| Total volume of infusion | 0.019 ± 0.002 | 0.111 | 0.524 |
| Duration of surgery | 0.015 ± 0.002 | 0.084 | 0.608 |
| Fever before surgery | 0.014 ± 0.001 | 0.080 | 0.688 |
| Total volume of fluid loss | 0.010 ± 0.002 | 0.059 | 0.747 |
| ASA | 0.010 ± 0.002 | 0.056 | 0.803 |
| Albumin | 0.008 ± 0.000 | 0.047 | 0.850 |
| Blood loss | 0.007 ± 0.001 | 0.038 | 0.888 |
| hs-CRP | 0.005 ± 0.000 | 0.029 | 0.917 |
| WBC | 0.005 ± 0.001 | 0.026 | 0.943 |
| ALT | 0.003 ± 0.000 | 0.016 | 0.959 |
| Creatinine | 0.003 ± 0.000 | 0.015 | 0.974 |
| Hemoglobin | 0.002 ± 0.000 | 0.011 | 0.985 |
| Age | 0.001 ± 0.000 | 0.006 | 0.991 |
| Gender | 0.001 ± 0.000 | 0.005 | 0.996 |
| History of smoking | 0.000 ± 0.000 | 0.002 | 0.998 |
| Diabetes mellitus | 0.000 ± 0.000 | 0.001 | 0.999 |
| Hypertension | 0.000 ± 0.000 | 0.001 | 1.000 |

Table S6. Logistic regression model performance in the training and validation cohort.

| **Variable** | **Training cohort** | **Validation cohort** |
| --- | --- | --- |
| AUC (95% CI) | 0.800 (0.787, 0.813) | 0.822 (0.790, 0.854) |
| Cutoff | 0.216 | 0.216 |
| Sensitivity | 0.718 | 0.739 |
| Specificity | 0.718 | 0.729 |
| Accuracy | 0.718 | 0.731 |
| Positive predictive value | 0.453 | 0.407 |
| Negative predictive value | 0.887 | 0.917 |

Table S7 Model effects of stratification analysis in the validation cohort.

| **Variables** | **N (%)** | **AUC** | **95% CI** | ***p-*value^*^** |
| --- | --- | --- | --- | --- |
| Total cohort | 1105 | 0.823 | 0.791-0.855 | - |
| Age (years) |  |  |  | 0.877 |
| 65-75 | 833 (75.40) | 0.830 | 0.792-0.868 |  |
| ≥75 | 272 (24.60) | 0.787 | 0.725-0.849 |  |
| Gender |  |  |  | 0.925 |
| Female | 445 (40.27) | 0.851 | 0.803-0.899 |  |
| Male | 660 (59.73) | 0.804 | 0.762-0.847 |  |
| Diabetes mellitus |  |  |  | 0.773 |
| No | 1021 (92.40) | 0.825 | 0.791-0.859 |  |
| Yes | 84 (7.60) | 0.781 | 0.671-0.891 |  |
| Hypertension |  |  |  | 0.633 |
| No | 550 (49.77) | 0.828 | 0.784-0.873 |  |
| Yes | 555 (50.23) | 0.817 | 0.771-0.862 |  |
| Blood loss (mL) |  |  |  | 0.888 |
| <100 | 724 (65.50) | 0.816 | 0.769-0.863 |  |
| ≥100 | 379 (34.30) | 0.772 | 0.719-0.825 |  |
| Type of surgery |  |  |  |  |
| Head and neck surgery | 101 (16.11) | 0.832 | 0.755-0.910 | ref. |
| Abdominal and pelvic surgery | 29 (4.63) | 0.682 | 0.429-0.934 | 0.867 |
| Skin, spine and joint surgery | 109 (17.38) | 0.634 | 0.517-0.752 | 0.997 |
| Cardiothoracic and vascular surgery | 312 (49.76) | 0.772 | 0.710-0.834 | 0.882 |
| Others | 76 (12.12) | 0.808 | 0.659-0.956 | 0.611 |

^*^Comparison of the area under the ROC curve for the two stratified populations using the z test.

**Figure S1. Cumulative weight of the variables and variable combination.**

**
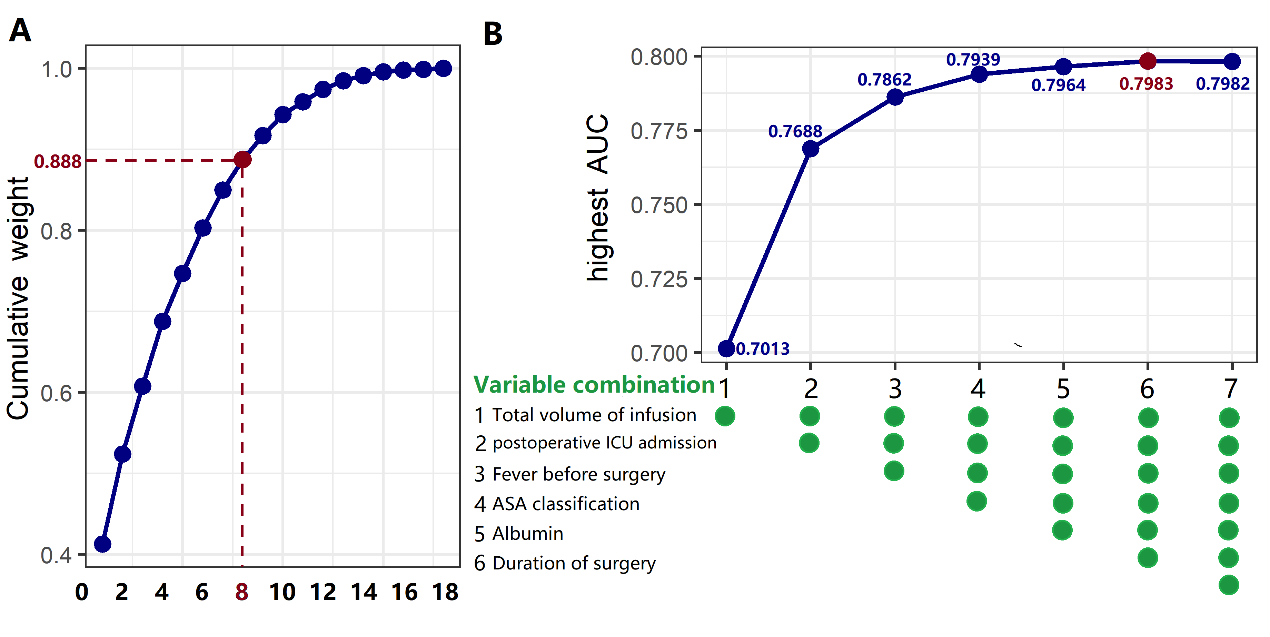
**

**Figure S2.** Correlation analysis of the continuous variables in the study.

**
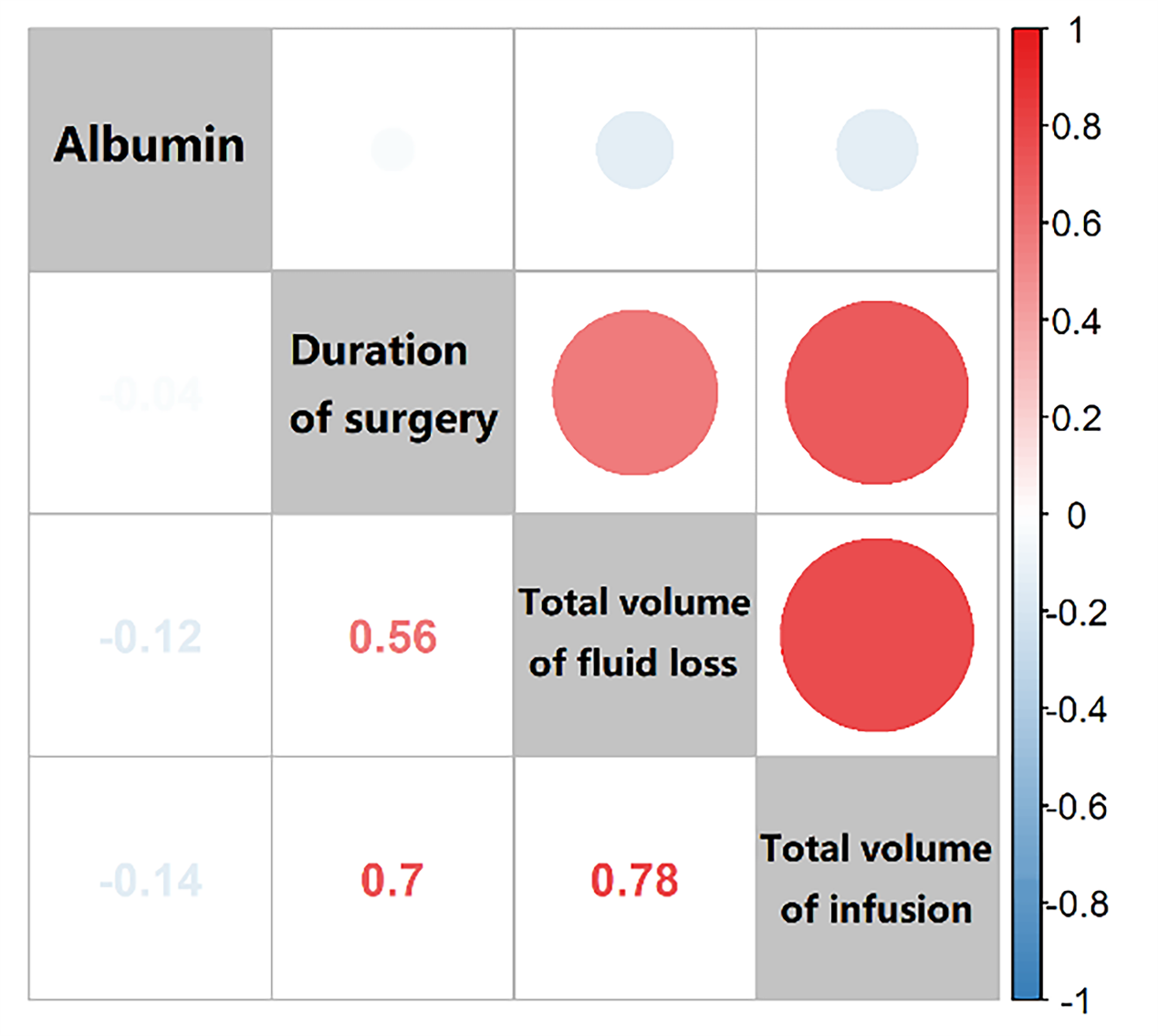
**

**Figure S3.** Decision curve analysis (DCA) of training cohort.

**
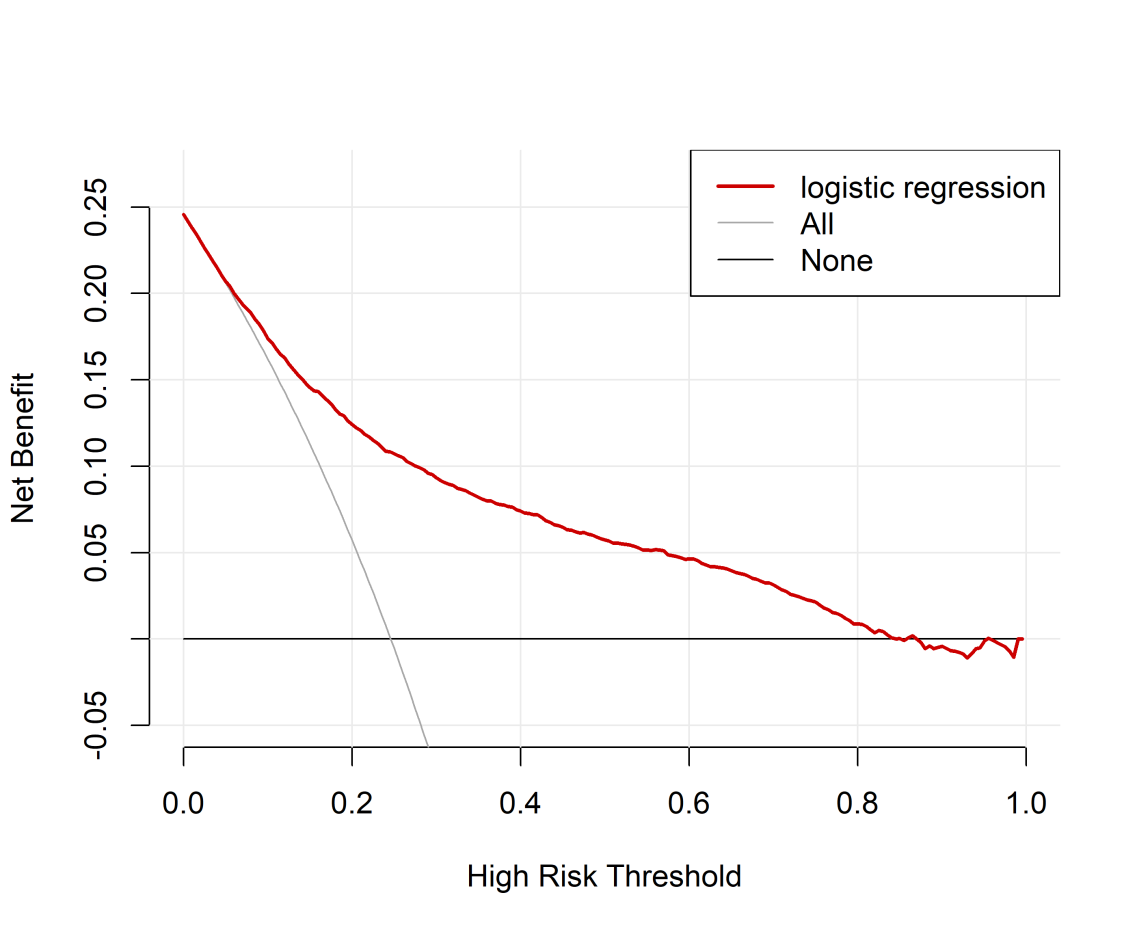
**
